# Diagnostic dilemma in a 3-year-old girl with acute nephritic syndrome and hematologic abnormalities: Answers

**DOI:** 10.1007/s00467-022-05752-6

**Published:** 2022-10-17

**Authors:** Samantha Innocenti, Silvia Bernardi, Maud Prévot, Antonin Saldmann, Maud Tusseau, Alexandre Belot, Jean-Paul Duong Van Huyen, Olivia Boyer

**Affiliations:** 1 Néphrologie Pédiatrique, Centre de Référence MARHEA, Hôpital Necker-Enfants Malades, APHP, Institut Imagine, Inserm U1163, Université Paris Cité, Paris, France; 2 Nephrology and Dialysis Unit, Meyer Children’s Hospital, Florence, Italy; 3 School of Nephrology, Università Degli Studi Di Milano, ASST Papa Giovanni XXIII, Bergamo, Italy; 4 Immunology Department, Hopital Européen Georges Pompidou, APHP, Cité University, Paris, Paris France; 5 Centre International de Recherche en Infectiologie, UMR5308, Univ Lyon Université Claude Bernard Centre National de La Recherche Scientifique ENS de Lyon, Lyon 1, U1111 InsermLyon, France; 6 Pediatric Nephrology, Dermatology Department, Hôpital Femme Mère Enfant, CRMR RAISE, Hospices Civils de Lyon, Rheumatology Bron, France; 7 The International Center of Research in Infectiology, UMR 5308, Lyon University CNRSENS, UCBL, INSERM U1111, Lyon, France; 8 Department of Pathology, Necker-Enfants Malades Hospital, APHP, Cité University, Paris, Paris France

**Keywords:** Monogenic lupus, Lupus nephritis, Childhood-onset systemic lupus erythematosus, Post-infectious glomerulonephritis

## Answers

**1) Which major diagnoses must be considered in this context?**

**Fig. 1:**
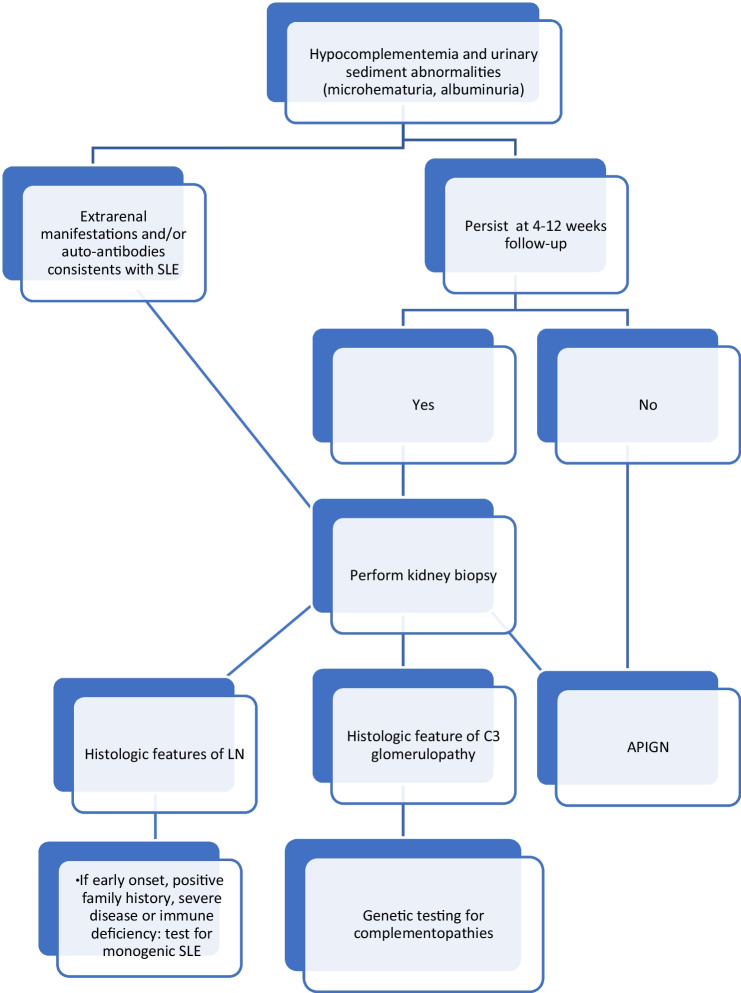
Simplified decisional tree for the initial evaluation of hypocomplementemia in children with kidney involvement. Mo, months; y, years

The first etiology to consider in the context of nephritic syndrome with low C3 level following a recent upper respiratory tract infection in an otherwise healthy 3-year-old girl is acute post-infectious glomerulonephritis (APIGN) (Fig. 1). APIGN is the most frequent cause of nephritic syndrome in childhood between 5 and 12 years of age. Clinical manifestations may vary from microscopic hematuria to full-blown acute nephritic syndrome with brown hematuria, proteinuria, edema, hypertension, and, in some cases, acute kidney injury (Eison et al. [1]). It usually occurs after group A beta-hemolytic streptococcal infection (pharyngitis or cutaneous involvement), but numerous other microbial agents have been associated with APIGN (Wenderfer [2]).

The time between the infection and the onset of nephritis depends on the site of infection and can range from 1 to 6 weeks. In addition to kidney involvement, laboratory tests usually show activation of the alternative pathway of complement (low circulating C3 and CH50) which should return to the normal range within 4 to 12 weeks after presentation. Diagnosis is based upon manifestations of acute nephritic syndrome and demonstration of recent streptococcal infection. Our group showed that a transient positivity of anti-factor B antibodies is associated with most APIGN cases in children and can guide the initial diagnostic approach with high sensitivity and specificity (Chauvet et al. [3]).

In case of persistent hypocomplementemia after 4 to 12 weeks, other causes of nephritic syndrome with low C3 levels must be ruled out by kidney histology, and a biopsy is therefore mandatory. They include membranoproliferative glomerulonephritis (MPGN) and lupus nephritis.

C3 glomerulonephritis (C3G) is a rare form of MPGN caused by complement alternative pathway dysregulation (Smith et al. [4]). This dysregulation may be associated with the presence of autoantibodies, such as C3 and C5 nephritic factor, anti-complement factor H, anti-complement factor B, and anti-C3b antibodies. Less frequently, genetic causes leading to an increased alternative pathway activation or an impaired regulation are identified. In some cases, both antibodies directed against complement regulatory proteins and genetic variants are noticed in the same patient confirming the complexity of the disease. The diagnosis of C3G is histopathological.

Immune complex-mediated MPGN can also be suspected. If confirmed, viral infective agents have to be screened such as HIV, HBV, and HCV. Even if HIV and hepatotropic viruses are infrequent causes of kidney disease in very young children in high-income countries, delayed diagnosis leads to unfavorable outcomes.

In pediatric HIV infection, kidney involvement is extremely rare (Bhimma et al. [5])and is more often associated with collapsing focal segmental glomerulosclerosis (FSGS) rather than immune mediated MPGN (Ramsuran et al. [6]).

In children with chronic HBV infection, extrahepatic manifestations are now very uncommon, and typical presentation is asymptomatic nephrotic range proteinuria with spontaneous remission (Bhimma and Coovadia [7]).

Even if MPGN is frequent in HCV chronic infection, the disease’s natural history has a slow progression so that the diagnosis of MPGN HCV-related in a 3-year-old girl is very unlikely.

Last but not least, the presence of an underlying autoimmune condition, especially childhood-onset systemic lupus erythematosus (cSLE), has to be investigated upon performing autoimmune screening. Systemic lupus erythematosus (SLE) is a rare disease under 5 years of age and can affect any organ or system with a pleomorphic presentation. Children may have an insidious and easily overlooked onset, with non-specific symptoms such as fever, modest arthralgias, and mild mucocutaneous complaints. Unfortunately, after onset, manifestations tend to be more severe than in adults. In fact, hematologic abnormalities, pleuritis, pericarditis (Hiraki et al. [8]), myopericardial disease (Chang et al. [9]), and kidney involvements are more common in children than in adults (Bundhun et al. [10]).

Furthermore, earlier onset, especially before 10 years, is also associated with life-threatening conditions such as macrophage activating syndrome, thrombosis, and neurological symptoms (Lopes [11])so that cSLE should also be suspected when these conditions occur. As in adults, the presence of antiphospholipid antibodies (aPL) heightens the overall thrombosis risk (Driest et al. [12]) and needs to be tested.

The 2019 EULAR/ACR classification criteria (Aringer et al. [13]) established ANA positivity (titer ≥ 1:80) as a mandatory criterion for diagnosis (Table1). Afterwards, some clinical and immunological criteria associated with kidney biopsy findings, each one specifically weighted, are evaluated to obtain a final score. If the patient’s total score is > 10, it can be classified as SLE. In cSLE, a higher cut-off (13 vs. 10) could improve the specificity of the classification (Rodrigues Fonseca et al. [14]). In this case, the patient had a total score > 20, only referring to clinical and immunological findings. Therefore, cSLE with kidney, hematological and mucocutaneous involvement was the most likely diagnosis.

**Table 1:** EULAR/ACR 2019 classification criteria. Modified from Aringer M et al. (Aringer et al. [13])

ANA positivity > 1/80			
Classified as SLE if EULAR/ACR score ≥ 10 with at least one clinical criterion			
Clinical domains	Weight (0–39 pt)	Immunology domains	Weight (0–12 pt)
Constitutional		Complement proteins	
Fever	2	Low C3 OR low C4	3
Hematologic		Low C3 AND low C4	4
Leucopenia	3	SLE-specific antibodies	
Thrombocytopenia	4	Anti-dsDNA OR anti-Smith antibody	6
Autoimmune hemolysis	4	Antiphospholipid antibodies	
Neuropsychiatric		Anti-cardiolipin OR antiβ2-GP1	2
Delirium	2	OR Lupus anticoagulant antibodies	
Psychosis	3		
Seizure	5		
Mucocutaneous			
Non-scarring alopecia	2		
Oral ulcer	2		
Subacute cutaneous or discoid lupus	4		
Acute cutaneous lupus	6		
Serosal			
Pleural or pericardial effusion	5		
Acute pericarditis	6		
Musculoskeletal			
Joint involvement	6		
Renal			
Proteinuria > 0.5 g/24 h	4		
Kidney biopsy class II or V	8		
Kidney biopsy class III or IV	10		

**2) What further investigations would you perform for the work-up of your main hypothesis?**

**Kidney biopsy**

The presence of extra-renal manifestations, a rapidly progressive glomerulonephritis, and/or a sustained hypocomplementemia for more than 8 to 12 weeks are strong indications to perform a kidney biopsy in the setting of acute nephritic syndrome in children. In the present patient, the indication emerged quickly as she presented severe hematological involvement and full-blown nephrotic syndrome.

**Fig. 2:**
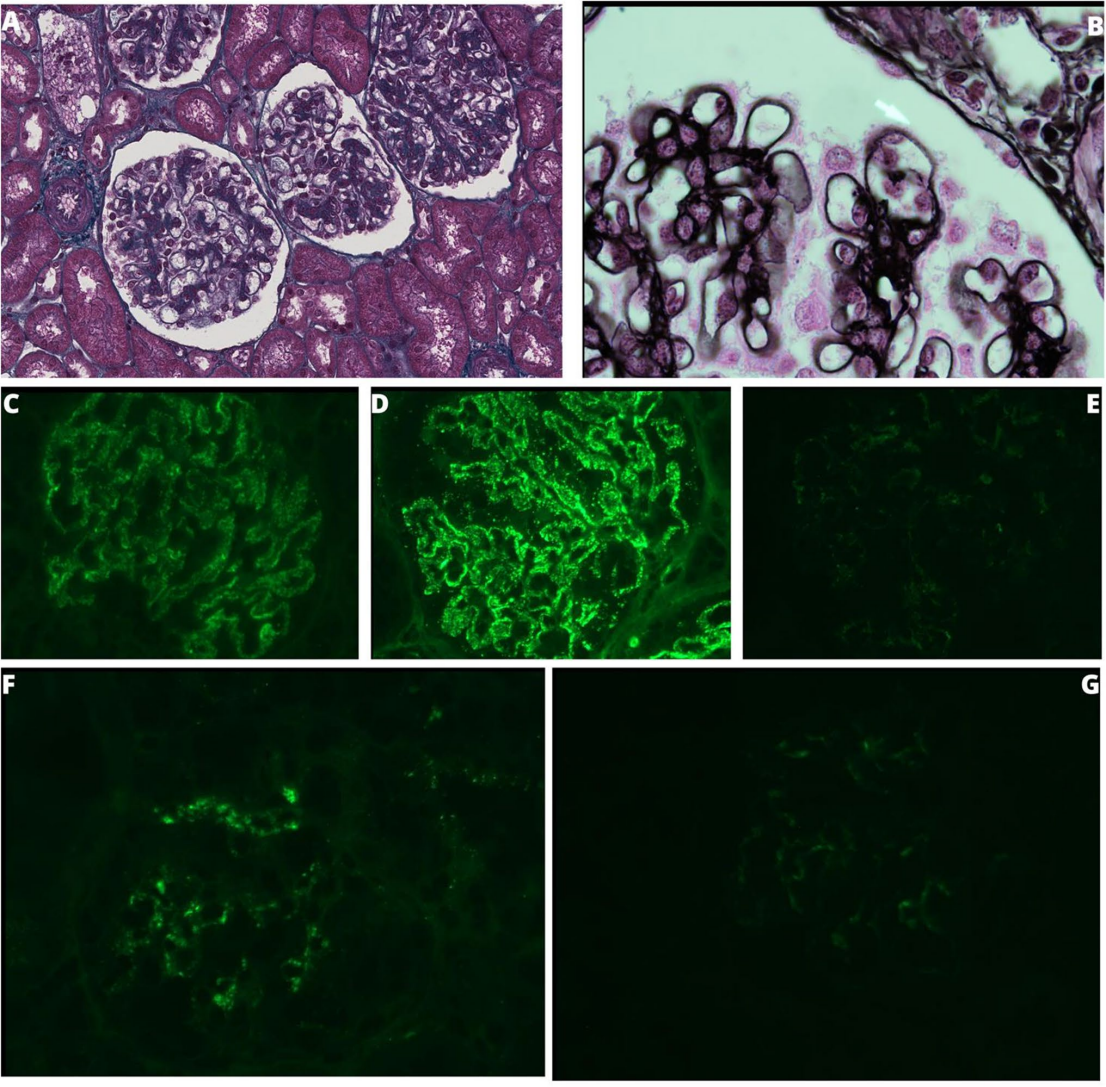
Histopathological findings on kidney biopsy. **A** Masson’s trichrome stain: diffuse glomerular hypertrophy and hyperplasia can be noticed without endo or extra-capillary proliferation. **B** Jones stain: some rare spikes of basement membrane (white arrow) are noticed. **C** Immunofluorescence staining for IgA: peripheral granular positivity (+ +). **D** Immunofluorescence staining for IgG: peripheral granular positivity (+ +). **E** Immunofluorescence staining for IgM: rare mesangial deposits. **F** Immunofluorescence staining for C3: mesangial positivity (+ +). **G** Immunofluorescence staining for C1q: peripheral positivity ( +). Conclusion: Lupus nephritis characterized by mild mesangial hypertrophy and hyperplasia with diffuse membranous immune deposits with full-house pattern highlighted in immunofluorescence. NIH modified score: activity 0/24, chronicity 0/12

In lupus nephritis (LN), biopsy and immunofluorescence findings are very characteristic (Bajema et al. [15]) and include glomerular deposits with the well-known “full-house” immunofluorescence pattern (predominant mesangial deposits of IgG and co-deposits of IgA, IgM, C3, and C1q) (Fig. 2). The glomerular lesions are then classified using the International Society of Nephrology/Renal Pathology Society Classification into proliferative (class II, III, and IV), non-proliferative lesions (class V), and advanced sclerosing lupus nephritis (class VI). The classification also provides information about severity and activity of the kidney disease as these indexes are probably related to kidney survival (Moroni [16]). Combining all of this information is essential in order to provide the best treatment regimen and tailored care.

Similarly, as for LN, identification of C3G is primarily based on immunofluorescence and after electron microscopy, as light microscopy features of membranoproliferative glomerulonephritis can be very heterogeneous. A predominant glomerular C3 staining is mandatory for the diagnosis, with electron-dense intramembranous deposits by electron microscopy.

**Genotyping**

**Fig. 3:**
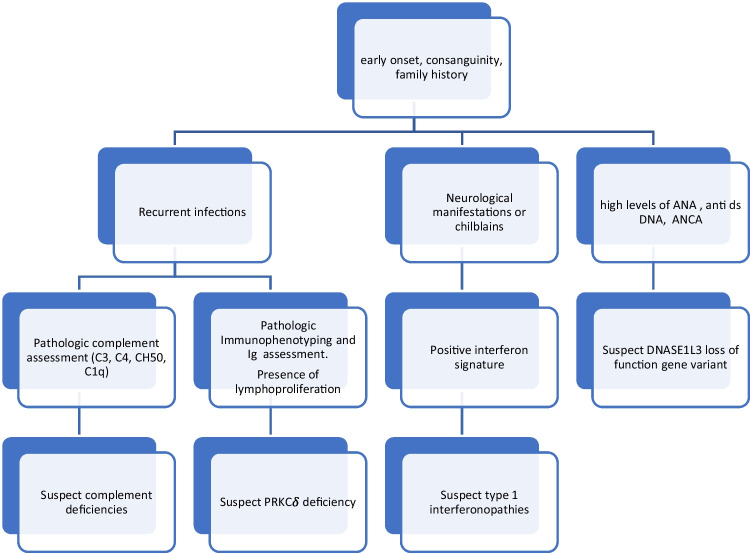
Initial evaluation based on clinical manifestations of cSLE. Ig, immunoglobulin; ANA, antinuclear antibodies; anti-dsDNA, anti-double-strand DNA antibodies; DNASE1L3, deoxyribonuclease 1 like 3; PRKC $\delta$, protein kinase C $\delta$

In newly diagnosed cSLE, a monogenic form of SLE should be suspected and furthermore investigated, especially in patients under 5 years of age with severe clinical manifestations (Lo [17]) (Fig. 3). Recent data report that monogenic SLEs are heterogeneous disorders involving various genes whose variants cause impaired immune tolerance (Demirkaya et al. [18]).

Genetic defects in the complement system were the first to be described. They often involve early complement proteins such as C1q, C1r/C1s, C4, or C2 deficiency, impairing opsonization and thereby leading to the accumulation of autoantigens.

Other important pathways implicated in the pathogenesis of monogenic SLE are type 1 interferonopathies (e.g., *STING*, *TREX1*) leading to the upregulation of interferon-stimulating genes and complement deficiencies. Defects in DNA damage repair (e.g., *TREX1*, DNase I), apoptosis (e.g., *FASLG*), and survival (e.g., *PRKCD*) of B lymphocytes, and clearance of self-antigen (*DNASE1L3*) are also implicated in loss of tolerance underlying the onset of monogenic SLE.

**Evaluation of extra-renal manifestations**

Extra-renal SLE manifestations should be investigated as well. As cardiac involvement may be silent in very young children, a trans-thoracic echocardiogram should be performed. In the same way, the execution of pulmonary function tests should be discussed as soon as the compliance of the patient improves enough, in order to identify a sub-clinical lung disease (Trapani et al. [19]).

**3) If the main hypothesis is confirmed, which treatment regimen could you consider?**

Following the EULAR/ACR 2019 guidelines, an induction therapy was performed with 3 boluses of methylprednisolone at 500 mg/m^2^/dose. After ophthalmologic evaluation, hydroxychloroquine was added. Subsequently, mycophenolate mofetil 500 mg twice a day and oral prednisone progressively tapered in 5 months were introduced as maintenance therapy.

## Discussion

Childhood-onset systemic lupus erythematosus is rare, especially before 5 years of age. Symptoms are commonly overlooked, despite the severity of certain clinical manifestations (Harry et al. [20]). Here, we present the case of a 3-year-old Guianese girl, with no past medical history, who presented acute nephritic syndrome following scarlet fever 2 months earlier and upper respiratory tract infections. Initial suspicion of APIGN was supported by the hypocomplementemia and recent infections. However, atypical features emerged with non-hemolytic anemia, positive direct Coombs test, thrombocytopenia, hypergammaglobulinemia, and high ESR:CRP ratio. Clinically, the patient presented a mild malar rash, palatal petechiae, and edema with rapid worsening of anemia and thrombocytopenia. Differential diagnosis then focused on viral infections or autoimmune diseases.

Autoimmune screen revealed high ANA, anti-dsDNA, ENA titer with anti-C1q, anti-platelet, anti-beta2GP1, and anti-phosphatidylserine/thrombin antibodies associated with LAC positivity, with no anti-FB antibodies or markers of macrophage activation. Coagulation profile was practically normal, without remarkable hypoprothrombinemia.

An active EBV replication was found but bone marrow aspiration and immunophenotyping excluded malignancies or EBV-induced cytopenia. Hence, although EBV infection may have played a role as a triggering cause of autoimmunity in this patient, it is not the leading cause of the kidney disease (Jog and James [21]).

Thus, in the setting of acute nephritic syndrome with autoimmunity, cSLE was highly plausible (Groot et al. [22]). Kidney biopsy was mandatory but had to be postponed due to the rapid worsening of clinical condition and thrombocytopenia. Thrombocytopenia is one the major bleeding risk factors after kidney biopsy, especially if the platelet count is under 120 × 10^3^μL. In cases of severe thrombocytopenia, to avoid platelet transfusions, immunosuppressive therapy may be started, and the kidney biopsy postponed until the platelet count has improved (Luciano and Moeckel [23]). Induction therapy with three methylprednisolone boluses and oral prednisone were administrated followed by maintenance therapy with mycophenolate and hydroxychloroquine. This led to a prompt normalization of the cell blood count enabling the kidney biopsy. Histologically, class V lupus nephritis with modified NIH-score of 0/24 for activity and 0/12 for chronicity was observed (Fig. 1).

An extensive examination of all phenotypes and the genetic abnormalities noticed in monogenic lupus is beyond the scope of this article, but concisely, the presence of concomitant chilblains, Aicardi–Goutières syndrome’s features, or immune-deficiency is highly suggestive for monogenic forms (Fig. 3).

In this patient, even if there was no familial history of SLE or consanguinity, the suspicion of an underlying monogenic form of cSLE was sustained by the very early onset, the severe phenotype with important cytopenias, and high levels of autoantibodies. Given the patient’s phenotype, a loss of function variant of *DNASE1L3*(Tusseau et al. [24]) or of C1q was suspected, but testing for this was negative. An extensive evaluation by whole-exome sequencing is still ongoing. Additionally, the apolipoprotein L1 (*APOL1*) G1 or G2 at-risk alleles for kidney diseases may accelerate progression to kidney failure in patients with LN (Hiraki [25]). Knowing the*APOL1* genotype could be useful to identify at-risk patients.

With this paper, we would like to raise awareness that even if APIGN is the leading cause of acute nephritis with low C3 levels in young children and kidney biopsy is not required in typical cases, there are some red flags suggesting differential diagnoses such as C3 glomerulopathy, membranoproliferative glomerulonephritis, or, as in this case, cSLE, requiring prompt and appropriate treatment and indicating a biopsy (Oni et al. [26]). These include sustained hypocomplementemia, rapidly progressive glomerulonephritis, or extra-renal manifestations (Fig. 1). Nowadays, more and more monogenic causes are identified in early-onset lupus nephritis, and genetic testing is recommended in young children or in case of suggestive features.
